# The Effects of Saline Water Drip Irrigation on Tomato Yield, Quality, and Blossom-End Rot Incidence --- A 3a Case Study in the South of China

**DOI:** 10.1371/journal.pone.0142204

**Published:** 2015-11-05

**Authors:** Yaming Zhai, Qian Yang, Maomao Hou

**Affiliations:** 1 Key Laboratory of Efficient Irrigation-Drainage and Agricultural Soil-Water Environment in Southern China (Hohai University), Ministry of Education, Nanjing, China; 2 College of Water Conservancy and Hydropower Engineering, Hohai University, Nanjing, China; 3 Henan Vocational College of Agriculture, Department of Gardening, Zhengzhou, China; 4 College of Horticulture, Fujian Agriculture and Forestry University, Fuzhou, Fujian, China; University of Vigo, SPAIN

## Abstract

Saline water resources are abundant in the coastal areas of south China. Most of these resources still have not been effectively utilized. A 3-year study on the effects of saline water irrigation on tomato yield, quality and blossom-end rot (BER) was conducted at different lower limits of soil matric potential (-10 kPa, -20 kPa, -30 kPa, -40 kPa and -50 kPa). Saline water differing in electrical conductivity (EC) (3 dS/m, 4 dS/m, 4.5 dS/m, 5 dS/m and 5.5 dS/m) was supplied to the plant after the seedling establishment. In all three years, irrigation water with 5.5 dS/m salinity reduced the maximum leaf area index (LAI_m_) and chlorophyll content the most significantly when compared with other salinity treatments. However, compared with the control treatment (CK), a slight increase in LAI_m_ and chlorophyll content was observed with 3~4 dS/m salinity. Saline water improved tomato quality, including fruit density, soluble solid, total acid, vitamin C and the sugar-acid ratio. There was a positive relationship between the overall tomato quality and salinity of irrigation water, as analyzed by principal component analysis (PCA). The tomato yield decreased with increased salinity. The 5.5 dS/m treatment reduced the tomato yield (Y_t_) by 22.4~31.1%, 12.6~28.0% and 11.7~27.3%, respectively in 2012, 2013 and 2014, compared with CK. Moreover, a significant (P≤0.01) coupling effect of salinity and soil matric potential on Y_t_ was detected. Saline water caused Y_t_ to increase more markedly when the lower limit of soil matric potential was controlled at a relatively lower level. The critical salinity level that produced significant increases in the BER_i_ was 3 dS/m~4 dS/m. Following the increase in BER_i_ under saline water irrigation, marketable tomato yield (Y_m_) decreased by 8.9%~33.8% in 2012, 5.1%~30.4% in 2013 and 10.1%~32.3% in 2014 compared with CK. In terms of maintaining the Y_t_ and Y_m_, the salinity of irrigation water should be controlled under 4 dS/m, and the lower limit of soil matric potential should be greater than -20 kPa.

## Introduction

In China, the imbalance between the supply and demand of freshwater resources is obvious, and the abundant saline water resources are not effectively utilized [1]. According to available statistics, China has 20 billion m^3^ of saline water every year, of which 13 billion m^3^ is available [2]. To address the shortage of freshwater and make full use of its saline water resources, China has applied various irrigation technologies to achieve water-efficient irrigation for agricultural systems for tomato crops. These technologies include direct irrigation with saline water [3,4], mixed irrigation with freshwater and saline water [5], and irrigation with freshwater and saline water in rotation [6]. Although saline water irrigation relieves shortages of water resources to various degrees, the improper use of saline water, such as the use of saline water with excessive salt content and the use of insufficient amounts of saline waters for irrigation, may cause both the water and the salinity stress, lead to a secondary salinization of cultivated soils, and bring a series of serious consequences to the agricultural environment and the ecosystem [7,8]. The soil matric potential is produced by the absorption and capillary forces in the soil. Irrigation to maintain a specified soil matric potential is commonly used in the production of many dry land crops. As a practical method, many scholars have studied controlling the threshold of soil matric potential for different crops under various soil and climate conditions [9,10,11]. The advantage of irrigation guided by considerations of soil matric potential is its regulatory effect on soil moisture that can create a comfortable growth environment for the roots of crops.

Under conditions of high soil salinity, many crop plants, including tomato, are susceptible and cannot survive or can survive only with decreased yields. To alleviate the deleterious effects of salinity, the measures such as the reclamation of salinized lands, the improvement of irrigation with saline water and the cultivation of salt-tolerant variety have been applied [12]. The most common one is the mixed irrigation with the freshwater and saline water. Several studies have specifically examined the effect of saline water irrigation on tomato growth, quality, yield and the incidence of blossom-end rot (BER_i_). Tomato organoleptic parameters such as soluble solids, fructose, glucose, titratable acid, and amino acid contents increase with increasing salinity [12,13,14,15,16,17]. As observed and considered in the context of the above factors, salt stress is applied to improve fruit quality, but little is known about the interaction between the organoleptic composition of tomato fruit and salt stress [18]. The positive changes in tomato quality have been obtained under certain salinity treatments. However, the tomato yield (Y_t_) has been reported to be negatively affected by the increasing salinity [19,20,21]. Furthermore, BER_i_ is known to be related to environmental factors, including high salinity [22,23]. BER is a major physiological disorder in tomato that produces losses of up to 50% [24], and the incidence of which is increased by salinity treatment aimed at increasing the soluble solid content of tomato [25].

Water is an important impact factor affecting the yield and quality of tomato crops. Higher levels of irrigation have been found to improve Y_t_, but decrease soluble sugar, organic acid and dry matter in the fruit [26]. The effect of deficit irrigation on fruit quality is generally the converse of the effect on fruit yield. Deficit irrigation has been shown to improve the total soluble solids content, titratable acidity and vitamin C content of tomato [27,28,29]. In north China, irrigation experiments controlled by lower limits on soil matric potential have shown that a -50 kPa lower limit was safe for the growth and yield of tomato [30].

Although many studies have investigated the agronomic performance of tomato individually under saline water irrigation and various soil moisture levels, few studies have looked into the combined effects of these factors on tomato growth, quality and yield. Moreover, in south China, whether irrigation by saline water threatens the normal output of tomato needs to be determined and subjected to further study. In the 3-year field experiment reported here, the growth responses of tomato to saline water irrigation and various levels of soil matric potential were compared and analyzed. In terms of crop performance, we hypothesized that all environmental factors have the same effects on the growth and development of tomato. The objectives of this study were to analyze the effect of saline water irrigation controlled by different lower limits of soil matric potential on tomato morphological index, quality, yield and BER_i_ and to evaluate better saline water irrigation methods with high utilization efficiencies of saline water while not significantly affecting the economic benefits of tomato production in south China. These treatments were compared with a freshwater irrigation treatment. All variables related to leaf area index (LAI), chlorophyll, quality, BER_i_, Y_t_ and marketable tomato yield (Y_m_) of tomatoes from each treatment were compared.

## Materials and Methods

### Experimental Conditions

**Fig 1:**
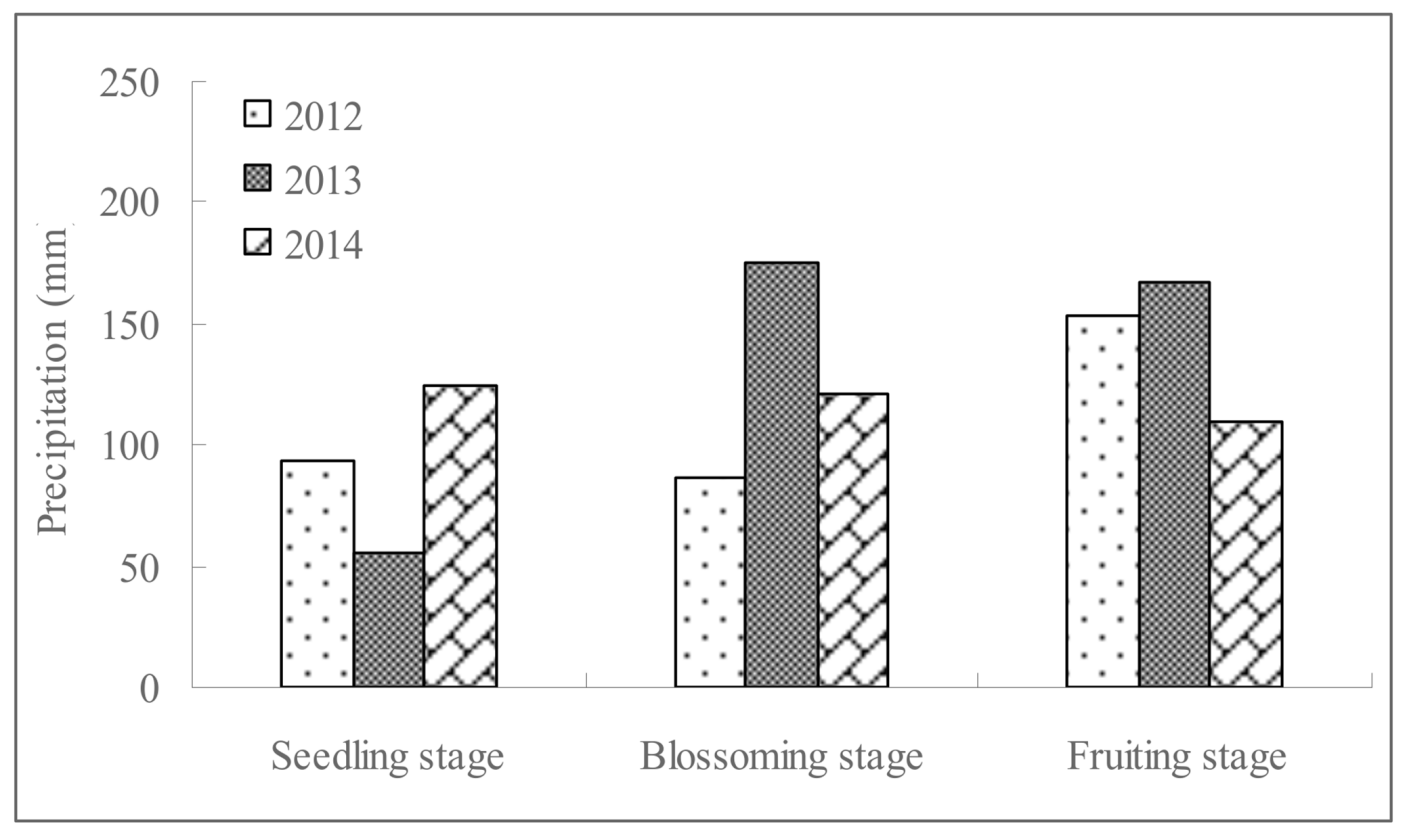
Precipitation in different tomato growth stages in 2012, 2013 and 2014.

The experiments were conducted for 3 consecutive years (2012~2014) at Modern Agricultural Park (permission to use the experimental fields was granted by the leading official responsible for the Park, and the study did not involve endangered and protected species) in Rudong County (E120°42′~121°22′, N32°12′~32°36′), south China. The coastline of Rudong County is 106 km in length with a beach area of 69,000 ha. Rudong County is in the subtropical maritime monsoon climate zone and is affected by the regulatory effects of the ocean and by monsoon circulation. The county enjoys clear seasons, a mild climate, abundant precipitation and sunlight. The perennial dominant wind in the county is a southeast wind with a mean wind speed of 3.5 m/s. The annual mean temperature of the experimental site is 15.0°C and the mean precipitation is 1042 mm, based on data from 1951~2002 [31]. The precipitation during the tomato growth season across the 3 study years is shown in Fig 1. The soil type in the experimental fields was ACfa (Alumi-Ferric Alisols) [32] with a pH of 7.4 and 1.6% organic matter. The soil bulk density for a depth of 0~60 cm was 1.38 g/cm^3^, which was the average value of 0~20 cm, 20~40 cm and 40~60 cm. The soil salt content was 1.1 g/kg. The EC of the soil solution was 0.4 dS/m. Moreover, the mean temperature during the entire growth stage of tomato was 22.9°C, 22.1°C and 23.9°C, respectively, in 2012, 2013 and 2014, and the mean humidity for each of these years was 72.8%, 77.2% and 78.1%, respectively.

### Experimental Design

Five saline water treatments (3 dS/m, 4 dS/m, 4.5 dS/m, 5 dS/m, 5.5 dS/m) were used to irrigate the tomato plants. Saline water treatments were prepared using the local subterranean freshwater (EC = 0.9 dS/m). Details of the saline water used for these treatments are shown in Table 1. The 5 lower-limit treatments of the soil matric potential were regulated below the 0.25 m depth of the water droppers. These values were -10 kPa, -20 kPa, -30 kPa, -40 kPa, -50 kPa. The irrigations were conducted once the soil matric potential decreased to the designation value. Five salinity treatments with irrigation water (W) combined with five lower-limit treatments of soil matric potential (M) resulted in a total of 25 treatments (WM). Irrigation treatments with freshwater served as control and were identified by the designation CK. Each treatment was replicated 3 times.

**Table 1:** Ionic composition of the saline water treatments.

EC/ dS/m	Ionic content (mmol/L)							
	Na^+^	K^+^	Mg^2+^	Ca^2+^	CO_3_^2-^	HCO_3_^-^	Cl^-^	SO_4_^2-^
0.9	2.4	0.6	3.8	0.5	0.5	5.8	2.4	1.5
3.0	13.8	3.9	7.5	2.6	0.5	14.2	9.5	6.0
4.0	20.7	4.9	7.8	1.8	0.5	18.8	13.1	8.1
4.5	23.2	5.7	9.0	2.1	0.5	21.1	15.4	9.1
5.0	26.1	6.6	10.2	2.4	0.5	23.4	17.8	9.8
5.5	28.7	7.1	10.9	2.7	0.5	25.3	19.4	10.6

### Agronomic Measures

Each year, only one season of tomato was cultivated. The tomato cultivar used was “*Red Crown*”. To provide the nutrients needed for the growth of the tomato plants, the experimental fields were fertilized with 650 kg/ha compound fertilizer (N: P_2_O_5_: K_2_O = 1:2:2) before transplanting.

Soil ridges were constructed to provide suitable growing conditions for the tomato plants. Each ridge was 60 cm wide. The ridges were spaced 100 cm apart. Two lines of tomato seedlings were transplanted on one ridge and spaced 40 cm apart. The experimental fields were blocked. A total of 12 tomato plants were planted in a 220 cm×60 cm block, and every 10 blocks were combined as one treatment for which the irrigation water and lower limit of soil matric potential were identical for the plants in this treatment. An impermeable membrane at a depth of 60 cm was used to separate different treatments and prevent the lateral seepage of irrigation water.

In mid-May in 2012~2014, after all experimental conditions were implemented, tomato seedlings with 6~8 leaves were transplanted to the fields. Conventional field management procedures were applied equally to all the seedlings. No additional light, heat, or CO_2_ were provided. The dates of transplanting, treating and first harvest during the 3 years are shown in Table 2.

**Table 2:** Dates of transplanting, treatment, first harvest, and harvest duration.

Year	Transplanting	Treatment	First harvesting	Harvest duration/d
2012	12-May	09-June (28DAT)	07-July (56DAT)	50
2013	16-May	18-June (33DAT)	22-July (67DAT)	52
2014	16-May	16-June (31DAT)	27-July (72DAT)	43

The lateral branches of the tomato plants were removed during the growth period, and topping treatments were applied in a timely manner. Each tomato plant was allowed to reserve 4 fruit sequences. Pest control was conducted according to the actual conditions occurring in the experimental fields.

### Irrigation and Top Application

Gravity drip irrigation was applied using casks with a volume of 100 L placed a distance of 1.5 m above the field. A drip pipe was arranged in the middle of the 2 rows of tomato plants. A water dropper interval of 20 cm was used. The average flow of the water dropper was 1 L/h.

After the tomato seedlings had been transplanted, 20 mm of fresh water (EC = 0.9 dS/m) was used for seedling survival. During the seedling survival stage, the lower limit of soil matric potential below the 0.25 m depth of the water droppers was -20 kPa. When the seedling survival stage was complete, the tomato plants were treated with saline water and a different lower limit of soil matric potential. Saline waters with different EC values were used to irrigate the tomato plants once the soil matric potential below 0.25 m depth of the water droppers decreased to the lower limit.

The 30% mass fraction solution of urea was applied as the additional fertilizer by adding it into the irrigation waters. The quantities of applied urea were 194 kg/ha, 232 kg/ha and 218 kg/ha in the 2012, 2013 and 2014 seasons, respectively.

### Measurements

Leaf area index (LAI): The LAI of the tomato plants was measured at every stage with a LAI 2000 Plant Canopy Analyzer (Li-Cor Biosciences USA).

Chlorophyll content: During the middle growth stage of the tomatoes, 6 representative plants were chosen from one treatment, and 5 upper functional leaves of each plant were collected for the measurement of chlorophyll content. The chlorophyll was extracted with a mixed liquor of acetone and absolute ethanol with a volume ratio of 1:1. Chlorophyll content was measured using the spectrophotometric method.

Y_t_ (t/a), BER_i_ (%) and Y_m_ (t/a): At the harvest stage, tomato fruits were picked manually every 3~5 days. The number and weight of both good fruit and the fruit with BER were recorded each time. Y_m_ were calculated as follows:

$$Y_{m}=Y_{t}\;-\;Y_{t}\times BER_{i}$$

Tomato quality: For each treatment, 20 tomato fruits that were red or orange in color were collected randomly for the measurement of the main physical and chemical components. The following components contributed greatly to the tomato taste and nutrient value: volume (*V*_*F*_), density (*ρ*_*F*_), soluble solids (*D*_*s*_), total acid (*G*), Vitamin C (*V*_*c*_) and sugar/acid ratio (*RSA*). The *V*_*F*_ was measured by the displacement method. *ρ*_*F*_ was calculated based on tomato volume and weight. The *D*_*s*_ was measured using a ACT-1E digital refractometer (ATAGO company, Japan). Total sugar was measured by the Fehling reagent titration method. *G* was measured by the sodium hydroxide titration method. The *V*_*c*_ content was measured by the 2, 6-dichloroindophenol titrimetric method [21].

### The PCA model

The principal component analysis (PCA) model was used to summarize the various quality indexes into one comprehensive quality index to facilitate the evaluation of tomato quality. The principal components of the quality indexes were extracted according to the following principles: “eigenvalue>1, cumulative contribution rate>80%”[33]. After the extraction, the value of the main component and the percentage contribution of that component to the total variation were obtained. The value of the comprehensive quality index was calculated using a membership function based on the value of the main component and percentage contribution. Detailed calculations are specified in [34].

### Data Analysis

The data were subjected to analysis of variance using a one-way ANOVA, and the means compared for statistical differences using the least significant difference (LSD) test at the 0.05 probability level in SPSS software Version 17.0 [21]. Data on the quality indexes for the PCA model were also submitted to the SPSS software to calculate the comprehensive quality index.

## Results

### The effect of different treatments on the maximum LAI (LAI_m_) of tomato

The LAI_m_ of tomatoes under different treatments across the 3 study years is shown in Table 3. Under a -10 kPa lower limit of soil matric potential, irrigation water with various salinities had no significant effects on LAI_m_, while under -20 ~ -50 kPa conditions, 5.5 dS/m salinity of irrigation water significantly (P≤0.05) reduced the LAI_m_ value, which was 17.1%, 15.9%, 12.5% and 11.8% lower compared to CK. The average LAI_m_ value first increased, then decreased with increasing salinity. The greatest average LAI_m_ value, 4.3, was found in both 3 dS/m and 4 dS/m salinity treatment for irrigation water. Among different lower limits of soil matric potential, except for the sharper decrease in LAI_m_ observed with -50 kPa (11.1% lower than average value), there were no obvious differences in LAI_m_ value for other treatments of soil matric potential. In the 2013 season, the LAI_m_ with different salinity treatments were 2.9~12.5% lower compared with CK. LAI_m_ still showed first an increase, then a decrease with increasing salinity. Similar to the 2012 season, the greatest LAI_m_ value of the 2013 season was found at 4 dS/m. The average LAI_m_ value with 4 dS/m salinity was 4.0. For the 2014 season, the LAI_m_ value presented a more dramatic decrease, which was 14.3~23.3% lower than that of 2012. The LAI_m_ value in 2014, 5.5 dS/m salinity, was lowest, 9.1% lower compared with that of CK. In contrast, in 2014, the LAI_m_ treated with 5.5 dS/m salinity and -50 kPa lower limit of soil matric potential (W5M5) was clearly lower than that of other treatments. Continuous cropping is well known to reduce LAI. Therefore, the lowest LAI_m_ with W5M5 might be explained by not only the higher salinity and lower irrigation amount but also the 3-year continuous cropping.

**Table 3:** The maximum leaf area index (LAI_m_) produced by tomato plants subjected to soil and water salinity treatments across the 3 years.

Year	Salinity(dS/m)	Soil matric potential (-kPa)					
		10	20	30	40	50	Average
2012	0.9 (CK)	4.0^a^	4.1^a^	4.4^a^	4.0^a^	3.4^b^	4.0
	3.0	4.4^a^	4.3^a^	4.5^a^	4.4^a^	3.8^ab^	4.3
	4.0	4.1^a^	4.4^a^	4.5^a^	4.2^a^	4.2^a^	4.3
	4.5	4.3^a^	4.0^a^	4.2^a^	3.8^a^	3.5^b^	4.0
	5.0	3.9^a^	3.7^a^	3.9^ab^	4.0^a^	3.7^ab^	3.8
	5.5	3.7^a^	3.4^b^	3.7^b^	3.5^b^	3.0^c^	3.5
	Average	4.1	4.0	4.2	4.0	3.6	4.0
2013	0.9 (CK)	3.5^b^	3.6^a^	3.3^b^	3.7^ab^	3.2^bc^	3.5
	3.0	4.0^a^	3.8^a^	3.7^a^	4.0^a^	3.8^a^	3.9
	4.0	3.7^ab^	4.0^a^	4.1^a^	4.3^a^	3.7^a^	4.0
	4.5	4.1^a^	3.5^a^	3.8^a^	4.1^a^	3.3^bc^	3.8
	5.0	4.2^a^	3.7^a^	3.0^b^	3.6^ab^	3.5^ab^	3.6
	5.5	3.4^b^	3.6^a^	3.3^b^	3.4^b^	3.1^c^	3.4
	Average	3.8	3.7	3.5	3.9	3.4	3.7
2014	0.9 (CK)	3.2^a^	3.4^a^	3.6^a^	3.3^ab^	3.1^ab^	3.3
	3.0	3.0^a^	3.3^a^	3.6^a^	3.6^a^	3.2^ab^	3.3
	4.0	3.5^a^	3.6^a^	3.5^a^	3.4^ab^	3.4^a^	3.5
	4.5	3.6^a^	3.3^a^	3.4^a^	3.3^ab^	3.1^ab^	3.3
	5.0	3.5^a^	3.1^a^	3.2^ab^	3.3^ab^	2.9^bc^	3.2
	5.5	3.3^a^	3.1^a^	2.9^b^	3.1^b^	2.7^c^	3.0
	Average	3.4	3.3	3.4	3.3	3.1	3.3

Note: The values of LAI_m_ in three years are means of 3 replications. In the same column and in the same year, means followed by the same letter (a, b) do not differ significantly at the 5% level by LSD.

### The effect of different treatments on tomato chlorophyll content

The leaf chlorophyll content of tomatoes during the 3 years was shown in Table 4. In 2012 season, the tomato chlorophyll content was affected by different salinity, and the greatest average value of 3.0 mg/g was discovered in 4 dS/m. Besides, different lower limits of soil matric potential also affected the chlorophyll content of tomatoes. Overall, chlorophyll content increased with decreases in the lower limit of soil matric potential, the greatest average value of 3.0 mg/g was obtained in the -50 kPa treatment. Compared with the 2012 season, the chlorophyll content for the 2013 season showed a decreasing trend, a decrease of 4.0~17.2% depending on the salinity of the irrigation water. The 3 dS/m, 4 dS/m and 4.5 dS/m salinity increased the chlorophyll content in 2013, but 5.5 dS/m significantly (P≤0.05) decreased the chlorophyll content at -20, -30 and -40 kPa condition, showing 12.50%, 10.00% and 16.7% decreases compared with that of CK. These results indicate that irrigation water with excessive salinity has negative effects on the chlorophyll content of tomato. The chlorophyll content of tomato in 2014 decreased compared with that in 2012 and 2013. This result might be also related to continuous cropping. In the 2014 season, the 4 dS/m increased the chlorophyll content of tomato most significantly. The chlorophyll content of tomato at 5.5 dS/m was lowest, and was only 1.7 mg/g. The pattern of influence of the lower limit of soil matric potential on chlorophyll content in 2014 was different from that in 2012 and 2013: the chlorophyll content of tomatoes in 2014 with -50 kPa was at a relatively lower level compared to that with other treatments of soil matric potential. According to the results from 2012~2014, the lower chlorophyll content found with -50 kPa might have resulted from the high salt residual in soils during the previous two years, the soil salts were not effectively eluted by irrigation waters. This damage in the growing environment of the tomatoes caused physiological changes in the tomato plants.

**Table 4:** The chlorophyll content in the leaf of tomato plants treated with water of different salinity during the 3 years (mg/g).

Year	Salinity (dS/m)	Soil matric potential (-kPa)					
		10	20	30	40	50	Average
2012	0.9 (CK)	2.2^b^	2.4^b^	2.4^c^	2.6^b^	2.7^b^	2.5
	3.0	2.9^a^	2.5^b^	2.7^bc^	2.5^b^	3.2^a^	2.8
	4.0	2.6^a^	2.9^a^	3.3^a^	3.0^a^	3.1^a^	3.0
	4.5	2.7^a^	2.6^b^	2.9^b^	2.9^a^	3.3^a^	2.9
	5.0	2.3^b^	2.4^b^	2.2^c^	2.6^b^	3.0^a^	2.5
	5.5	2.1^b^	2.4^b^	2.4^c^	2.5^b^	2.8^b^	2.4
	Average	2.5	2.5	2.7	2.7	3.0	2.7
2013	0.9 (CK)	1.9^c^	2.4^a^	2.0^c^	2.4^b^	2.5^b^	2.2
	3.0	2.3^b^	2.6^a^	2.3^b^	2.5^b^	2.7^ab^	2.5
	4.0	2.7^a^	2.3^ab^	2.6^a^	2.9^a^	2.9^a^	2.7
	4.5	2.0^c^	2.3^ab^	2.5^a^	2.8^a^	2.4^b^	2.4
	5.0	2.3^b^	2.5^a^	2.4^ab^	2.3^b^	2.3^b^	2.4
	5.5	2.0^c^	2.1^b^	1.8^d^	2.0^c^	2.3^b^	2.0
	Average	2.2	2.4	2.3	2.5	2.5	2.4
2014	0.9 (CK)	1.8^b^	1.8^b^	2.3^b^	2.2^b^	2.0^b^	2.0
	3.0	2.2^a^	2.3^a^	2.3^b^	2.4^a^	2.2^a^	2.3
	4.0	2.3^a^	2.2^a^	2.7^a^	2.5^a^	2.4^a^	2.4
	4.5	2.0^ab^	2.0^ab^	2.2^b^	2.2^b^	2.3^a^	2.1
	5.0	1.8^b^	2.0^ab^	2.0^c^	1.8^c^	1.9^b^	1.9
	5.5	1.6^c^	1.8^b^	1.7^d^	1.8^c^	1.4^c^	1.7
	Average	2.0	2.0	2.2	2.2	2.0	2.1

Note: The values of chlorophyll content in three years are means of 3 replications. In the same column and in the same year, means followed by the same letter (a, b) do not differ significantly at the 5% level by LSD.

### The effect of different treatments on tomato quality

Table 5 shows the main quality indexes observed over the 3 years. The quality indexes in the table are the means from the five soil matric potential treatments. In all 3 years, the higher salinity of irrigation water increased *ρ*_*F*_, but these differences were not statistically significant. *V*_*F*_ was significantly affected by the salinity of the irrigation water (P≤0.05). In 2012 the irrigation treatments with 4 dS/m, 4.5 dS/m, 5 dS/m and 5.5 dS/m salinity decreased the *V*_*F*_ value (P≤0.05). This decrease was also found with 5 dS/m and 5.5 dS/m salinity in 2013 and with 4.5 dS/m, 5 dS/m and 5.5 dS/m salinity in 2014. Therefore, the results showed that the average volume of a single tomato fruit would dramatically decrease if the salinity of irrigation water was more than 5 dS/m.

**Table 5:** The quality indexes of tomatoes with different treatments across the 3 years.

Year	Salinity (dS/m)	Density/ *ρ*_*F*_ (g/cm^3^)	Volume/ *V*_*F*_ (cm^3^)	Soluble solid/ *D*_*S*_ (%)	Total acid/ *G* (g/100 g)	Vitamin C/ *V*_*C*_ (mg/100 g)	Sugar/acid ratio (*RSA*)
2012	0.9 (CK)	0.927±0.011^a^	143.1±3.1^a^	5.0±0.2^c^	0.52±0.01^c^	10.0±0.6^e^	7.3±0.2^e^
	3.0	0.935±0.006^a^	135.6±3.3^ab^	5.2±0.4^c^	0.55±0.01^c^	11.2±0.5^de^	7.7±0.4^de^
	4.0	0.936±0.004^a^	131.1±3.7^bc^	5.9±0.4^b^	0.54±0.03^c^	11.7±0.6^cd^	8.4±0.2^cd^
	4.5	0.938±0.005^a^	133.3±3.7^bc^	6.2±0.2^ab^	0.61±0.03^b^	12.7±0.5^bc^	8.9±0.2^bc^
	5.0	0.939±0.006^a^	130.7±2.3^bc^	6.4±0.3^ab^	0.65±0.02^ab^	13.5±0.8^ab^	9.3±0.5^ab^
	5.5	0.943±0.008^a^	126.1±4.3^c^	6.8±0.3^a^	0.69±0.03^a^	14.3±0.5^a^	9.9±0.3^a^
	Average	0.936	133.3	5.9	0.59	12.2	8.6
2013	0.9 (CK)	0.931±0.002^a^	140.0±7.8^a^	4.7±0.3^d^	0.53±0.02^c^	9.9±0.4^d^	8.0±0.6^d^
	3.0	0.938±0.004^a^	136.3±5.0^ab^	5.0±0.3^cd^	0.54±0.02^c^	11.2±0.6^c^	8.8±0.4^cd^
	4.0	0.933±0.008^a^	133.0±5.9^abc^	5.5±0.2^c^	0.55±0.03^c^	13.8±0.5^b^	9.4±0.3^bc^
	4.5	0.938±0.006^a^	129.3±3.4^abc^	6.4±0.2^b^	0.61±0.01^b^	12.8±0.6^b^	9.8±0.5^b^
	5.0	0.941±0.010^a^	127.1±2.0^bc^	6.8±0.3^ab^	0.63±0.03^ab^	14.9±0.3^a^	10.3±0.4^ab^
	5.5	0.940±0.022^a^	122.9±3.8^c^	7.3±0.3^a^	0.67±0.03^a^	15.4±0.4^a^	10.8±0.1^a^
	Average	0.937	131.4	5.9	0.59	13.0	9.5
2014	0.9 (CK)	0.935±0.005^a^	140.7±8.1^a^	4.7±0.4^d^	0.50±0.03^e^	9.4±0.6^c^	7.2±0.4^d^
	3.0	0.947±0.015^a^	133.5±4.2^ab^	4.8±0.3^d^	0.53±0.02^de^	10.2±0.2^bc^	9.0±0.5^bc^
	4.0	0.943±0.005^a^	131.2±5.9^ab^	5.5±0.2^cd^	0.58±0.06^cd^	10.7±0.6^b^	8.3±0.3^c^
	4.5	0.945±0.011^a^	128.3±4.7^bc^	6.0±0.5^bc^	0.64±0.03^bc^	11.9±0.4^a^	9.5±0.3^ab^
	5.0	0.945±0.016^a^	126.0±1.8^bc^	6.6±0.3^ab^	0.69±0.02^ab^	12.6±0.5^a^	9.8±0.4^a^
	5.5	0.954±0.003^a^	118.6±3.2^c^	7.1±0.1^a^	0.72±0.02^a^	12.8±0.6^a^	10.3±0.2^a^
	Average	0.945	129.7	5.8	0.61	11.3	9.0

Note: The values of the quality indexes in the three years are means of 3 replications. In the same column and in the same year, means followed by the same letter (a, b) do not differ significantly at the 5% level according to an LSD test. Each value is the mean ± SD (n = 3).

The *D*_*s*_ increased as the salinity of the irrigation water increased. In 2012 and 2013, the *D*_*s*_ value was enhanced by a salinity of more than 4 dS/m (P≤0.05). Similarly, the *D*_*s*_ value was significantly enhanced by a salinity of more than 4.5 dS/m in 2014 (P≤0.05). A salinity of 5.5 dS/m increased the *D*_*s*_ value most significantly, by 38.2%, 54.2% and 51.2% compared with that of CK across the 3 years. Similar to *D*_*s*_, *G* was also positively affected by the higher salinity of irrigation waters, but *G* did not differ significantly between CK and 3 dS/m. In all 3 years, *G* was increased by salinities of 4.5 dS/m, 5 dS/m and 5.5 dS/m (P≤0.05), the sharpest increase of which was found with 5.5 dS/m salinity in 2013, 54.3% greater than that of CK. However, in this study, a higher *G* value was not beneficial for the evaluation of the comprehensive quality of tomato because a higher *G* would affect the taste of the tomato fruit. Therefore, during the quality evaluation process, G was a “the lower the better” index.

The salinity of the irrigation water positively affected the *V*_*C*_ content. In 2012 and 2014, irrigation water with salinity other than 3 dS/m increased the *V*_*C*_ value significantly (P≤0.05). In 2013, all the saline water irrigation treatments increased the *V*_*C*_ value significantly (P≤0.05). Of all the salinity treatments, 5.5 dS/m increased the *V*_*C*_ value most dramatically, by 42.4%, 56.3% and 36.5%, respectively, compared with that of CK. Overall, the *RSA* increased as salinity increased across the 3 years. During 2012 and 2013, *RSA* did not differ significantly between CK and 3 dS/m, but in 2014, the *RSA* with 3 dS/m salinity was higher than that with CK (P≤0.05). In all 3 years, a salinity greater than 4 dS/m would significantly increase *RSA*. The greatest increase in *RSA*, compared with CK, was observed with 5.5 dS/m salinity. These increases were 34.9%, 35.3% and 43.4%, respectively, in the 3 years.

**Fig 2:**
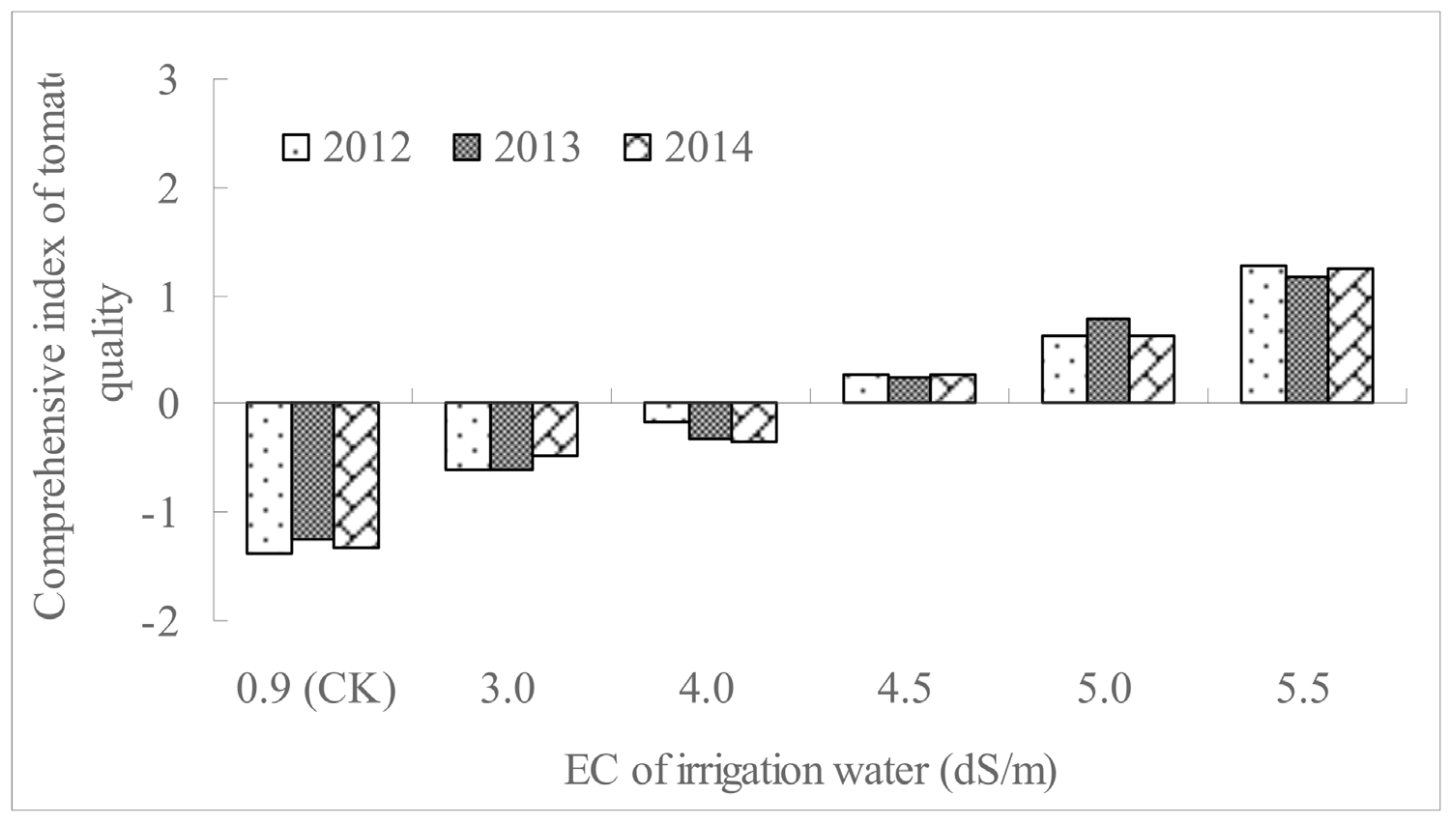
The comprehensive quality index (CQI) of tomato fruits irrigated using saline waters with different EC across the 3 years (The CQI was calculated using the Principle Component Analysis).

The comprehensive quality index of tomato fruit with different saline water treatments, calculated by PCA, across the 3 years is shown in Fig 2. A higher comprehensive quality index indicated a better comprehensive quality (the 6 indexes observed) of tomato [35]. The results of the present study show that higher-salinity irrigation water produced tomatoes of better comprehensive quality.

### The effect of different treatments on Y_t_ and BER_i_

Table 6 shows the yield of tomatoes under different treatments across the 3 years. It was clear that Y_t_ with 5.5 dS/m treatment was lower than that of other salinity treatments. Compared to CK, 5.5 dS/m treatment reduced Y_t_ by 22.4~31.1%, 12.6~28.0% and 11.7~27.3%, respectively in 2012, 2013 and 2014. A salinity of more than 5 dS/m significantly (P≤0.05) reduced Y_t_, but there was no significant difference in yield between 3 dS/m and CK. In terms of the average value of yield across the 3 years, irrigation water with a salinity of more than 3 dS/m but lower than 4 dS/m generally ensured a normal yield.

**Table 6:** Tomato yield (Y_t_) with different treatments across the 3 years (t/ha).

Year	Salinity (dS/m)	Soil matric potential (-kPa)					
		10	20	30	40	50	Average
2012	0.9 (CK)	111.9±6.9^a^	133.4±4.2^a^	127.5±3.8^a^	113.9±8.8^a^	94.2±9.7^a^	116.2
	3.0	97.2±3.4^ab^	130.1±3.6^ab^	120.8±2.1^a^	108.4±3.3^ab^	93.7±3.1^ab^	110.0
	4.0	106.7±7.0^a^	118.3±4.7^bc^	120.8±5.0^a^	117.3±6.2^a^	89.7±8.3^a^	110.6
	4.5	103.1±6.4^a^	108.8±4.8^cd^	101.3±5.2^b^	97.6±6.0^bc^	85.4±4.4^bc^	99.2
	5.0	86.2±5.2^bc^	104.2±6.4^d^	96.1±3.2^bc^	89.4±2.0^cd^	81.9±9.7^cd^	91.6
	5.5	80.8±7.8^c^	103.5±7.8^d^	87.9±3.2^c^	82.7±3.9^d^	71.2±2.8^d^	85.2
	Average	97.6	116.4	109.1	101.6	86.0	102.1
2013	0.9 (CK)	100.5±4.6^ab^	136.8±4.5^a^	123.5±3.0^a^	113.2±4.9^ab^	103.0±6.7^a^	115.4
	3.0	104.5±10.6^a^	129.4±2.9^a^	125.6±4.9^a^	124.0±5.5^a^	97.5±6.3^ab^	116.2
	4.0	108.9±4.2^a^	127.6±5.4^a^	114.8±3.9^b^	110.8±4.3^ab^	97.5±9.3^a^	111.9
	4.5	99.0±5.9^abc^	113.8±5.4^b^	102.2±2.9^c^	107.0±5.8^bc^	85.5±3.6^bc^	101.5
	5.0	85.6±3.3^c^	101.8±5.6^c^	98.8±3.6^cd^	94.7±5.4^cd^	79.6±7.5^cd^	92.1
	5.5	87.8±5.0^bc^	98.5±4.7^c^	93.1±4.7^d^	90.4±9.8^d^	76.6±4.7^d^	89.3
	Average	97.7	118.0	109.7	106.7	90.0	104.4
2014	0.9 (CK)	105.8±3.2^a^	130.0±2.7^a^	118.7±4.0^a^	102.2±2.3^ab^	98.3±10.6^ab^	111.0
	3.0	104.3±4.1^ab^	120.9±3.8^a^	107.7±3.1^b^	97.7±5.8^ab^	103.3±4.7^ab^	106.8
	4.0	110.3±7.3^a^	104.2±3.3^b^	102.1±3.1^b^	107.6±5.2^a^	96.4±5.2^a^	104.1
	4.5	100.2±5.2^ab^	95.6±9.6^b^	100.3±2.8^bc^	97.5±5.7^ab^	89.7±3.5^ab^	96.7
	5.0	87.6±3.4^c^	92.0±6.8^b^	92.7±2.3^cd^	92.5±4.4^bc^	82.3±3.3^bc^	89.4
	5.5	93.4±5.7^bc^	94.5±6.7^b^	88.9±6.2^d^	83.9±6.1^c^	65.6±6.4^c^	85.3
	Average	100.3	106.2	101.8	96.9	89.3	98.9
W	78.737**						
M	95.864**						
W*M	2.410**						

Note: The values of Y_t_ in three years are means of 3 replications. In the same column and in the same year, means followed by the same letter (a, b) do not differ significantly at the 5% level according to a LSD test. Each value is the mean ± SD (n = 3). Figures prior to ** were the F values, and ** mean significance at P≤0.01.

The soil matric potential treatments also affected Y_t_. In the same year, the -10 kPa and -50 kPa soil matric potential limits resulted in relatively low Y_t_ values. A possible reason for this finding is that the soil moisture at a -10 kPa lower limit represented a higher level that harmed the growth and development of tomatoes, whereas a -50 kPa lower limit would create water stress for the tomato plants. A -50 kPa limit of soil matric potential reduced Y_t_ most dramatically, on average 26.1%, 23.7% and 15.9% lower than the highest yield (with -20 kPa lower limit) in 2012, 2013 and 2014, respectively. Treatments with a -20 kPa lower limit produced the highest Y_t_ and were, on average, 116.4 t/ha, 118.0 t/ha and 106.2 t/ha in 2012, 2013 and 2014, respectively.

Based on the above findings, it was recommended that irrigation water with a salinity of lower than 4 dS/m combined with a -20 kPa lower limit of soil matric potential be considered optimal for obtaining a higher yield of tomato.

**Fig 3:**
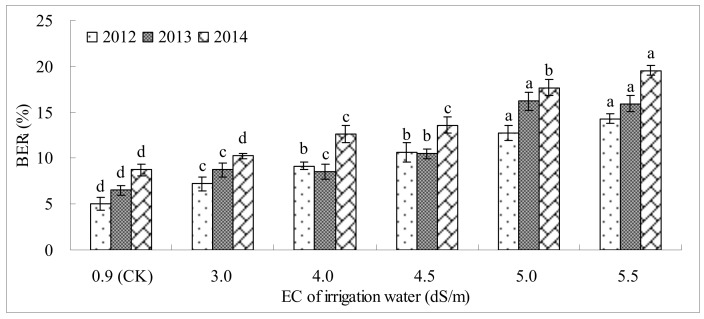
The incidence of blossom-end rot (BER_i_) of tomato irrigated with saline waters of different electrical conductivity (EC) values across the 3 years. (At the same EC of irrigation water, the values of tomato BER incidence are the means from the five soil matric potential treatments. In the same year, means followed by the same letter do not differ significantly at the 5% level according to a LSD test. Each value is the mean ± SD).

Fig 3 shows the BER_i_ of tomatoes irrigated using saline waters with different EC values across the 3 years. In 2012, BER_i_ values ranged from 5.0%~14.3%. Irrigation treatments with 3~5.5 dS/m salinity significantly (P≤0.05) increased BER_i_ compared with CK, and BER_i_ with 5.5 dS/m was 2.84 times greater than BER_i_ with CK. However, the BER_i_ values at 4 dS/m, 4.5 dS/m, 5 dS/m and 5.5 dS/m did not differ significantly. Likewise, BER_i_ increased with higher salinity in 2013 and 2014. The salinity of 5.5 dS/m increased BER_i_ most dramatically and was 2.45 times and 2.24 times greater than that with CK in 2013 and 2014, respectively. Moreover, it was found that BER_i_ increased overall over the study years. BER_i_ was clearly greater in 2014 than in 2012 and 2013. Most likely, this result is explained by the negative effects of continuous cropping.

**Fig 4:**
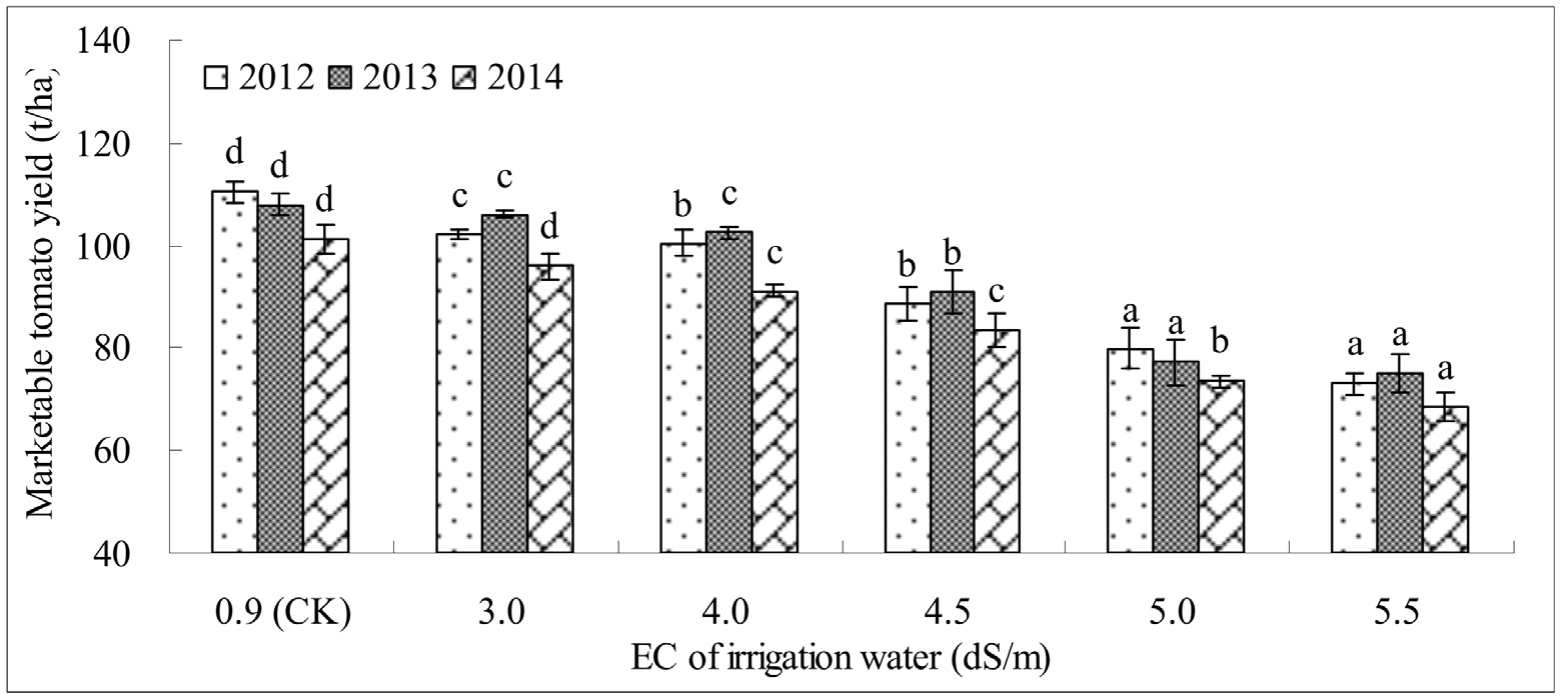
The marketable yield (Y_m_) of tomato irrigated with saline waters of different EC across the 3 years (Under the same EC of irrigation water, values of Y_m_ are the means of the five soil matric potential treatments. In the same year, means followed by the same letter do not differ significantly at the 5% level according to a LSD test. Each value is the mean ± SD).

Fig 4 shows the Y_m_ of tomato irrigated using saline waters with different EC levels across the 3 study years. In 2012, Y_m_ was significantly less with saline water treatments than with CK. A level of 5.5 dS/m decreased Y_m_ markedly, but Y_m_ did not differ significantly (P≥0.05) between 3 dS/m and 4 dS/m. In 2013, Y_m_ also decreased at 3 dS/m and 4 dS/m, but Y_m_ at 3 dS/m and 4 dS/m did not differ significantly from Y_m_ at CK. Irrigation water with a salinity greater than 4.5 dS/m significantly (P≤0.05) decreased Y_m_ in 2013. In 2014, as in 2012, Y_m_ decreased with increases in salinity. Y_m_ decreased most markedly at 5.5 dS/m. At this level, it was 32.3% lower than that at CK.

## Discussion

Irrigated agriculture will continue to play a significant role in producing the world’s food supply. Approximately 30~40% of the world’s food is produced by irrigated farmlands, which represent only 16% of the world’s total agricultural area [36]. In China, 81% of the total freshwater resources occur in south China, whereas 64% of the farmland occurs in north China [37]. For irrigated agriculture to be sustainable, the utilization of water resources must be highly efficient. At present, there are no generally accepted design criteria for saline water management in humid areas. Studies have shown that improper irrigation by saline water will lead to a high accumulation of salts in the ploughed layer, impairing the growth environment for crops [8,38]. Thus, there is a pressing need to develop new management methods for saline water utilization to meet the challenge of a sustainable irrigated agriculture that conserves water resources and has a minimum impact on crop growth and development. The current practice of rotating irrigation between saline water and non-saline water has been shown to decrease the damage to crops produced by salt, relative to crops irrigated directly with saline water.

In general, salt in irrigation waters (especially NaCl) will result in pernicious effects on crops, such as decreased turgor pressure, a lowered speed of cell expansion and damage to chloroplasts, thus reducing the growth rate and photosynthesis. These changes ultimately influence the dry matter accumulation and yield of crops. Salinity stress has been reported to show an oxidative component that is present due to the uncontrolled generation of reactive oxygen species and damage to the antioxidative system [39]. Alarcon’s [40] study showed that a decrease in relative growth rate and leaf area ratio in tomato plants in response to increasing osmotic pressure of the nutrient solution could be detected within an experimental period of 17 days. Karin’s [41] study demonstrated that a slight salt stress improves osmotic adjustment, enhances adenosine triphosphatase enzymatic activity and stimulates crop growth. In this study, under saline water irrigation, although saline water with a salinity of less than 4 dS/m had no obvious impact on tomato LAI_m_ and chlorophyll content, decreases of LAI_m_ by 2.86%~12.50% and chlorophyll content by 4.00%~15.0% were found at 5.5 dS/m salinity. This result supports the findings of Zhang [42], who noted that saline water of more than 3 g/L NaCl decreased LAI and chlorophyll. Under irrigation with saline water of various salinity levels, -30 kPa and -40kPa lower limits of soil matric potential were more beneficial for tomato in maintaining normal levels of LAI_m_ and chlorophyll content.

Under the high-saline water irrigation (5.5 dS/m) treatment, the fruit soluble solids, total acid, vitamin C and sugar-acid ratio increased by 38.2~54.2%, 25.9~43.1%, 36.5~50.2% and 34.9~43.3%, respectively. A positive relationship between the tomato comprehensive quality and salinity of irrigation water was found in the experiment. In both 3-year periods, irrigation water with 5.5 dS/m salinity improved the tomato quality markedly. A similar study by Zushi [18] showed that salt enhanced tomato sensory attributes as a result of increases in sugar, organic acid, and amino acid contents. Beckles [43] noted that increasing the electrical conductivity (EC) of soil, either by applying a high ionic solution or by restricting watering, stimulated an increase in sugar concentration per fruit. The mechanism of the increased soluble sugar content under saline water irrigation had been studied by Lu [25], who found that moderate salt stress enhanced the activity of sucrose invertase.

Under saline water irrigation, a decrease in fruit yield corresponds to a decrease in fruit weight and in the number of fruit [44]. The results of the current study also indicated that saline water irrigation decreased volume per fruit. Moreover, irrigation water with more than 5 dS/m salinity decreased Y_t_ significantly (P≤0.05) with a maximum rate of decrease of 31.1%, 28.0% and 33.3% in 2012, 2013 and 2014, respectively, but there was no significant difference in yield between 3 dS/m and CK. A similar study by Cramer [45] showed that where electrical conductivity (EC) levels were increased beyond 0.4 S m^-1^ by the addition of major nutrients (N, K^+^, Mg^2+^ and Ca^2+^), the fruit had lower yields and a higher content of dry matter. Kamaluldeen [46] noted that water salinity was more injurious to Y_t_ than soil salinity. Moreover, we detected a significant (P≤0.01) coupling effect of salinity and soil matric potential on Y_t_. This result confirmed many other studies showing that tomato is sensitive to water salinity, particularly when hot and water stresses also occur [47,48,49]. In this study, the salinity-induced yield reduction might have been due to a decreased inflow of water into the fruits [50].

In addition to taste and flavor, BER is important for tomato marketability. BER_i_ may be induced by stresses in the root zone, e.g., salinity, soil water stress, NH^4+^ toxicity and oxygen withholding [22,51]. Tabatabaie [52] noted that a nutrient solution with high EC (6 dS/m) would increase BER_i_ dramatically. In our study, saline water with as little as 4 dS/m significantly increased BER_i_, and BER_i_ was positively related to the salinity of irrigation water. Irrigation water with 4~5.5 dS/m salinity increased BER_i_ by 4.1%~9.3% in 2012, 2.0%~9.4% in 2013 and 3.8%~10.8% in 2014. This change led to a decrease of Y_m_ to various degrees in all 3 years (by 8.9%~33.8% in 2012, 5.1%~30.4% in 2013, and 10.1%~32.3% in 2014). BER is considered a calcium-deficit induced physiological disorder [19]. The reason for this increased BER_i_ might be that under conditions of high soil salinity, it was difficult for calcium to move from the tomato root to the fruit [53]. Besides, it should also be mentioned that the blossom end rot incidence is often lower in soils with pH from 6–6.9, and that it is associated with high N applications [54].

Saline-water irrigation increases salt accumulation in the plough layer. An experiment conducted in northwest China found that the salinity of the original soils (The soil bulk density varies from 1.27 to 1.45 mg/m^3^ in the 1 m soil profile, with a pH range of 7.50~7.88. The average electrical conductivity of the soil saturation extract in the soil profile varies from 1.15 to 1.90 dS/m) increased 447% when irrigated with high-saline water [1]. However, the current study only investigated the effect on tomatoes of saline-water irrigation controlled with different lower limits of soil matric potential. This study did not investigate how the treatments affected the distribution of soil salt accumulation. More research on this topic needs to be conducted in the future.

In this study, saline water irrigation controlled by lower limit of soil matric potential furnished a new method for the study of tomato irrigation. The objectives of this new management approach were to safely utilize saline water resources that maintain economic yield and improve the quality of tomato. Note, however, that this experiment was conducted in south China. The characteristics of saline water, climate and soil conditions might be different in other places. In practice, the relationship between soil moisture and soil matric potential can be quantified. The soil matric potential could be managed by frequently regulating soil moisture.

## Supporting Information

S1 Fig Precipitation in different tomato growth stages in 2012, 2013 and 2014. (DOC)

S2 Fig The comprehensive quality index (CQI) of tomato fruits irrigated using saline waters with different EC across the 3 years (The CQI was calculated using the Principle Component Analysis). (DOC)

S3 Fig The incidence of blossom-end rot (BER_i_) of tomato irrigated with saline waters of different electrical conductivity (EC) values across the 3 years. (At the same EC of irrigation water, the values of tomato BER incidence are the means from the five soil matric potential treatments. In the same year, means followed by the same letter do not differ significantly at the 5% level according to a LSD test. Each value is the mean ± SD).
(DOC)

S4 Fig The marketable yield (Y_m_) of tomato irrigated with saline waters of different EC across the 3 years (Under the same EC of irrigation water, values of Y_m_ are the means of the five soil matric potential treatments. In the same year, means followed by the same letter do not differ significantly at the 5% level according to a LSD test. Each value is the mean ± SD).
(DOC)

S1 Table Ionic composition of different saline water treatments. (DOC)

S2 Table The maximum leaf area index (LAI_m_) produced by tomato plants subjected to soil and water salinity treatments across the 3 years. (DOC)

S3 Table The chlorophyll content in the leaf of tomato plants treated with water of different salinity during the 3 years (mg/g). (DOC)

S4 Table The quality indexes of tomatoes with different treatments across the 3 years. (DOC)

S5 Table Tomato yield (Y_t_) with different treatments across the 3 years (t/ha). (DOC)
