# Supplementary material for: The Effects of Saline Water Drip Irrigation on Tomato Yield, Quality, and Blossom-End Rot Incidence --- A 3a Case Study in the South of China
Source: PLoS One. 2015 Nov 5;10(11):e0142204. doi: 10.1371/journal.pone.0142204 (PMC4634986; doi:10.1371/journal.pone.0142204)
Supplement: S1 Table — (DOC) [file pone.0142204.s005.doc]

| EC/ dS/m | Ionic content (mmol/L) | | | | | | | |
| --- | --- | --- | --- | --- | --- | --- | --- | --- |
|  | Na^+^ | K^+^ | Mg^2+^ | Ca^2+^ | CO_3_^2-^ | HCO_3_^-^ | Cl^-^ | SO_4_^2-^ |
| 0.9 | 2.4 | 0.6 | 3.8 | 0.5 | 0.5 | 5.8 | 2.4 | 1.5 |
| 3 | 13.8 | 3.9 | 7.5 | 2.6 | 0.5 | 14.2 | 9.5 | 6.0 |
| 4 | 20.7 | 4.9 | 7.8 | 1.8 | 0.5 | 18.8 | 13.1 | 8.1 |
| 4.5 | 23.2 | 5.7 | 9.0 | 2.1 | 0.5 | 21.1 | 15.4 | 9.1 |
| 5 | 26.1 | 6.6 | 10.2 | 2.4 | 0.5 | 23.4 | 17.8 | 9.8 |
| 5.5 | 28.7 | 7.1 | 10.9 | 2.7 | 0.5 | 25.3 | 19.4 | 10.6 |
