# Supplementary material for: The Effects of Saline Water Drip Irrigation on Tomato Yield, Quality, and Blossom-End Rot Incidence --- A 3a Case Study in the South of China
Source: PLoS One. 2015 Nov 5;10(11):e0142204. doi: 10.1371/journal.pone.0142204 (PMC4634986; doi:10.1371/journal.pone.0142204)
Supplement: S2 Table — (DOC) [file pone.0142204.s006.doc]

| Year | Salinity  (dS/m) | Soil matric potential (-kPa) | | | | | |
| --- | --- | --- | --- | --- | --- | --- | --- |
|  |  | 10 | 20 | 30 | 40 | 50 | Average |
| 2012 | 0.9 (CK) | 4.0^a^ | 4.1^a^ | 4.4^a^ | 4.0^a^ | 3.4^b^ | 4.0 |
|  | 3 | 4.4^a^ | 4.3^a^ | 4.5^a^ | 4.4^a^ | 3.8^ab^ | 4.3 |
|  | 4 | 4.1^a^ | 4.4^a^ | 4.5^a^ | 4.2^a^ | 4.2^a^ | 4.3 |
|  | 4.5 | 4.3^a^ | 4.0^a^ | 4.2^a^ | 3.8^a^ | 3.5^b^ | 4.0 |
|  | 5 | 3.9^a^ | 3.7^a^ | 3.9^ab^ | 4.0^a^ | 3.7^ab^ | 3.8 |
|  | 5.5 | 3.7^a^ | 3.4^b^ | 3.7^b^ | 3.5^b^ | 3.0^c^ | 3.5 |
|  | Average | 4.1 | 4.0 | 4.2 | 4.0 | 3.6 | 4.0 |
| 2013 | 0.9 (CK) | 3.5^b^ | 3.6^a^ | 3.3^b^ | 3.7^ab^ | 3.2^bc^ | 3.5 |
|  | 3 | 4.0^a^ | 3.8^a^ | 3.7^a^ | 4.0^a^ | 3.8^a^ | 3.9 |
|  | 4 | 3.7^ab^ | 4.0^a^ | 4.1^a^ | 4.3^a^ | 3.7^a^ | 4.0 |
|  | 4.5 | 4.1^a^ | 3.5^a^ | 3.8^a^ | 4.1^a^ | 3.3^bc^ | 3.8 |
|  | 5 | 4.2^a^ | 3.7^a^ | 3.0^b^ | 3.6^ab^ | 3.5^ab^ | 3.6 |
|  | 5.5 | 3.4^b^ | 3.6^a^ | 3.3^b^ | 3.4^b^ | 3.1^c^ | 3.4 |
|  | Average | 3.8 | 3.7 | 3.5 | 3.9 | 3.4 | 3.7 |
| 2014 | 0.9 (CK) | 3.2^a^ | 3.4^a^ | 3.6^a^ | 3.3^ab^ | 3.1^ab^ | 3.3 |
|  | 3 | 3.0^a^ | 3.3^a^ | 3.6^a^ | 3.6^a^ | 3.2^ab^ | 3.3 |
|  | 4 | 3.5^a^ | 3.6^a^ | 3.5^a^ | 3.4^ab^ | 3.4^a^ | 3.5 |
|  | 4.5 | 3.6^a^ | 3.3^a^ | 3.4^a^ | 3.3^ab^ | 3.1^ab^ | 3.3 |
|  | 5 | 3.5^a^ | 3.1^a^ | 3.2^ab^ | 3.3^ab^ | 2.9^bc^ | 3.2 |
|  | 5.5 | 3.3^a^ | 3.1^a^ | 2.9^b^ | 3.1^b^ | 2.7^c^ | 3.0 |
|  | Average | 3.4 | 3.3 | 3.4 | 3.3 | 3.1 | 3.3 |
