# Supplementary material for: The Effects of Saline Water Drip Irrigation on Tomato Yield, Quality, and Blossom-End Rot Incidence --- A 3a Case Study in the South of China
Source: PLoS One. 2015 Nov 5;10(11):e0142204. doi: 10.1371/journal.pone.0142204 (PMC4634986; doi:10.1371/journal.pone.0142204)
Supplement: S3 Table — (DOC) [file pone.0142204.s007.doc]

| Year | Salinity  (dS/m) | Soil matric potential (-kPa) | | | | | |
| --- | --- | --- | --- | --- | --- | --- | --- |
|  |  | 10 | 20 | 30 | 40 | 50 | Average |
| 2012 | 0.9 (CK) | 2.2^b^ | 2.4^b^ | 2.4^c^ | 2.6^b^ | 2.7^b^ | 2.5 |
|  | 3 | 2.9^a^ | 2.5^b^ | 2.7^bc^ | 2.5^b^ | 3.2^a^ | 2.8 |
|  | 4 | 2.6^a^ | 2.9^a^ | 3.3^a^ | 3.0^a^ | 3.1^a^ | 3.0 |
|  | 4.5 | 2.7^a^ | 2.6^b^ | 2.9^b^ | 2.9^a^ | 3.3^a^ | 2.9 |
|  | 5 | 2.3^b^ | 2.4^b^ | 2.2^c^ | 2.6^b^ | 3.0^a^ | 2.5 |
|  | 5.5 | 2.1^b^ | 2.4^b^ | 2.4^c^ | 2.5^b^ | 2.8^b^ | 2.4 |
|  | Average | 2.5 | 2.5 | 2.7 | 2.7 | 3.0 | 2.7 |
| 2013 | 0.9 (CK) | 1.9^c^ | 2.4^a^ | 2.0^c^ | 2.4^b^ | 2.5^b^ | 2.2 |
|  | 3 | 2.3^b^ | 2.6^a^ | 2.3^b^ | 2.5^b^ | 2.7^ab^ | 2.5 |
|  | 4 | 2.7^a^ | 2.3^ab^ | 2.6^a^ | 2.9^a^ | 2.9^a^ | 2.7 |
|  | 4.5 | 2.0^c^ | 2.3^ab^ | 2.5^a^ | 2.8^a^ | 2.4^b^ | 2.4 |
|  | 5 | 2.3^b^ | 2.5^a^ | 2.4^ab^ | 2.3^b^ | 2.3^b^ | 2.4 |
|  | 5.5 | 2.0^c^ | 2.1^b^ | 1.8^d^ | 2.0^c^ | 2.3^b^ | 2.0 |
|  | Average | 2.2 | 2.4 | 2.3 | 2.5 | 2.5 | 2.4 |
| 2014 | 0.9 (CK) | 1.8^b^ | 1.8^b^ | 2.3^b^ | 2.2^b^ | 2.0^b^ | 2.0 |
|  | 3 | 2.2^a^ | 2.3^a^ | 2.3^b^ | 2.4^a^ | 2.2^a^ | 2.3 |
|  | 4 | 2.3^a^ | 2.2^a^ | 2.7^a^ | 2.5^a^ | 2.4^a^ | 2.4 |
|  | 4.5 | 2.0^ab^ | 2.0^ab^ | 2.2^b^ | 2.2^b^ | 2.3^a^ | 2.1 |
|  | 5 | 1.8^b^ | 2.0^ab^ | 2.0^c^ | 1.8^c^ | 1.9^b^ | 1.9 |
|  | 5.5 | 1.6^c^ | 1.8^b^ | 1.7^d^ | 1.8^c^ | 1.4^c^ | 1.7 |
|  | Average | 2.0 | 2.0 | 2.2 | 2.2 | 2.0 | 2.1 |
