# Supplementary material for: The Effects of Saline Water Drip Irrigation on Tomato Yield, Quality, and Blossom-End Rot Incidence --- A 3a Case Study in the South of China
Source: PLoS One. 2015 Nov 5;10(11):e0142204. doi: 10.1371/journal.pone.0142204 (PMC4634986; doi:10.1371/journal.pone.0142204)
Supplement: S4 Table — (DOC) [file pone.0142204.s008.doc]

| Year | Salinity  (dS/m) | *ρ_F_*  (g/cm^3^) | *V_F_*  (cm^3^) | *D_S_*  (%) | *G*  (g/100 g) | *V_C_*  (mg/100 g) | *RSA* |
| --- | --- | --- | --- | --- | --- | --- | --- |
| 2012 | 0.9 (CK) | 0.927±0.011^a^ | 143.05±3.12^a^ | 4.95±0.15^c^ | 0.524±0.014^c^ | 10.04±0.59^e^ | 7.30±0.22^e^ |
|  | 3 | 0.935±0.006^a^ | 135.57±3.28^ab^ | 5.24±0.35^c^ | 0.554±0.007^c^ | 11.17±0.50^de^ | 7.73±0.36^de^ |
|  | 4 | 0.936±0.004^a^ | 131.14±3.74^bc^ | 5.91±0.35^b^ | 0.540±0.031^c^ | 11.74±0.58^cd^ | 8.40±0.17^cd^ |
|  | 4.5 | 0.938±0.005^a^ | 133.26±3.67^bc^ | 6.21±0.19^ab^ | 0.606±0.027^b^ | 12.66±0.45^bc^ | 8.94±0.24^bc^ |
|  | 5 | 0.939±0.006^a^ | 130.74±2.29^bc^ | 6.36±0.32^ab^ | 0.645±0.018^ab^ | 13.48±0.77^ab^ | 9.31±0.48^ab^ |
|  | 5.5 | 0.943±0.008^a^ | 126.06±4.31^c^ | 6.84±0.30^a^ | 0.687±0.029^a^ | 14.29±0.46^a^ | 9.85±0.33^a^ |
| 2013 | 0.9 (CK) | 0.931±0.002^a^ | 140.04±7.76^a^ | 4.72±0.33^d^ | 0.532±0.021^c^ | 9.87±0.37^d^ | 7.98±0.56^d^ |
|  | 3 | 0.938±0.004^a^ | 136.29±5.02^ab^ | 4.98±0.31^cd^ | 0.542±0.016^c^ | 11.22±0.60^c^ | 8.80±0.38^cd^ |
|  | 4 | 0.933±0.008^a^ | 132.95±5.87^abc^ | 5.45±0.24^c^ | 0.553±0.027^c^ | 13.77±0.50^b^ | 9.42±0.34^bc^ |
|  | 4.5 | 0.938±0.006^a^ | 129.32±3.39^abc^ | 6.43±0.16^b^ | 0.606±0.013^b^ | 12.76±0.61^b^ | 9.79±0.51^b^ |
|  | 5 | 0.941±0.010^a^ | 127.05±2.02^bc^ | 6.79±0.31^ab^ | 0.631±0.028^ab^ | 14.94±0.30^a^ | 10.25±0.38^ab^ |
|  | 5.5 | 0.940±0.022^a^ | 122.91±3.77^c^ | 7.28±0.26^a^ | 0.670±0.033^a^ | 15.42±0.39^a^ | 10.80±0.07^a^ |
| 2014 | 0.9 (CK) | 0.935±0.005^a^ | 140.72±8.13^a^ | 4.71±0.44^d^ | 0.504±0.026^e^ | 9.37±0.59^c^ | 7.21±0.35^d^ |
|  | 3 | 0.947±0.015^a^ | 133.47±4.22^ab^ | 4.83±0.29^d^ | 0.534±0.017^de^ | 10.23±0.16^bc^ | 8.95±0.50^bc^ |
|  | 4 | 0.943±0.005^a^ | 131.18±5.92^ab^ | 5.47±0.23^cd^ | 0.582±0.055^cd^ | 10.73±0.58^b^ | 8.33±0.25^c^ |
|  | 4.5 | 0.945±0.011^a^ | 128.27±4.65^bc^ | 5.96±0.51^bc^ | 0.636±0.026^bc^ | 11.93±0.41^a^ | 9.54±0.34^ab^ |
|  | 5 | 0.945±0.016^a^ | 125.99±1.84^bc^ | 6.56±0.27^ab^ | 0.694±0.020^ab^ | 12.63±0.49^a^ | 9.79±0.39^a^ |
|  | 5.5 | 0.954±0.003^a^ | 118.59±3.22^c^ | 7.11±0.13^a^ | 0.721±0.024^a^ | 12.79±0.63^a^ | 10.33±0.18^a^ |
