# Supplementary material for: The Effects of Saline Water Drip Irrigation on Tomato Yield, Quality, and Blossom-End Rot Incidence --- A 3a Case Study in the South of China
Source: PLoS One. 2015 Nov 5;10(11):e0142204. doi: 10.1371/journal.pone.0142204 (PMC4634986; doi:10.1371/journal.pone.0142204)
Supplement: S5 Table — (DOC) [file pone.0142204.s009.doc]

| Year | Salinity  (dS/m) | Soil matric potential (-kPa) | | | | | |
| --- | --- | --- | --- | --- | --- | --- | --- |
|  |  | 10 | 20 | 30 | 40 | 50 | Average |
| 2012 | 0.9 (CK) | 111.9±6.9^a^ | 133.4±4.2^a^ | 127.5±3.8^a^ | 113.9±8.8^a^ | 94.2±9.7^a^ | 116.2 |
|  | 3 | 97.2±3.4^ab^ | 130.1±3.6^ab^ | 120.8±2.1^a^ | 108.4±3.3^ab^ | 93.7±3.1^ab^ | 110.0 |
|  | 4 | 106.7±7.0^a^ | 118.3±4.7^bc^ | 120.8±5.0^a^ | 117.3±6.2^a^ | 89.7±8.3^a^ | 110.6 |
|  | 4.5 | 103.1±6.4^a^ | 108.8±4.8^cd^ | 101.3±5.2^b^ | 97.6±6.0^bc^ | 85.4±4.4^bc^ | 99.2 |
|  | 5 | 86.2±5.2^bc^ | 104.2±6.4^d^ | 96.1±3.2^bc^ | 89.4±2.0^cd^ | 81.9±9.7^cd^ | 91.6 |
|  | 5.5 | 80.8±7.8^c^ | 103.5±7.8^d^ | 87.9±3.2^c^ | 82.7±3.9^d^ | 71.2±2.8^d^ | 85.2 |
|  | Average | 97.6 | 116.4 | 109.1 | 101.6 | 86.0 | 102.1 |
| 2013 | 0.9 (CK) | 100.5±4.6^ab^ | 136.8±4.5^a^ | 123.5±3.0^a^ | 113.2±4.9^ab^ | 103.0±6.7^a^ | 115.4 |
|  | 3 | 104.5±10.6^a^ | 129.4±2.9^a^ | 125.6±4.9^a^ | 124.0±5.5^a^ | 97.5±6.3^ab^ | 116.2 |
|  | 4 | 108.9±4.2^a^ | 127.6±5.4^a^ | 114.8±3.9^b^ | 110.8±4.3^ab^ | 97.5±9.3^a^ | 111.9 |
|  | 4.5 | 99.0±5.9^abc^ | 113.8±5.4^b^ | 102.2±2.9^c^ | 107.0±5.8^bc^ | 85.5±3.6^bc^ | 101.5 |
|  | 5 | 85.6±3.3^c^ | 101.8±5.6^c^ | 98.8±3.6^cd^ | 94.7±5.4^cd^ | 79.6±7.5^cd^ | 92.1 |
|  | 5.5 | 87.8±5.0^bc^ | 98.5±4.7^c^ | 93.1±4.7^d^ | 90.4±9.8^d^ | 76.6±4.7^d^ | 89.3 |
|  | Average | 97.7 | 118.0 | 109.7 | 106.7 | 90.0 | 104.4 |
| 2014 | 0.9 (CK) | 105.8±3.2^a^ | 130.0±2.7^a^ | 118.7±4.0^a^ | 102.2±2.3^ab^ | 98.3±10.6^ab^ | 111.0 |
|  | 3 | 104.3±4.1^ab^ | 120.9±3.8^a^ | 107.7±3.1^b^ | 97.7±5.8^ab^ | 103.3±4.7^ab^ | 106.8 |
|  | 4 | 110.3±7.3^a^ | 104.2±3.3^b^ | 102.1±3.1^b^ | 107.6±5.2^a^ | 96.4±5.2^a^ | 104.1 |
|  | 4.5 | 100.2±5.2^ab^ | 95.6±9.6^b^ | 100.3±2.8^bc^ | 97.5±5.7^ab^ | 89.7±3.5^ab^ | 96.7 |
|  | 5 | 87.6±3.4^c^ | 92.0±6.8^b^ | 92.7±2.3^cd^ | 92.5±4.4^bc^ | 82.3±3.3^bc^ | 89.4 |
|  | 5.5 | 93.4±5.7^bc^ | 94.5±6.7^b^ | 88.9±6.2^d^ | 83.9±6.1^c^ | 65.6±6.4^c^ | 85.3 |
|  | Average | 100.3 | 106.2 | 101.8 | 96.9 | 89.3 | 98.9 |
| W | 78.737** | | | | | | |
| M | 95.864** | | | | | | |
| W*M | 2.410** | | | | | | |
