# Supplementary material for: The Effects of Saline Water Drip Irrigation on Tomato Yield, Quality, and Blossom-End Rot Incidence --- A 3a Case Study in the South of China
Source: PLoS One. 2015 Nov 5;10(11):e0142204. doi: 10.1371/journal.pone.0142204 (PMC4634986; doi:10.1371/journal.pone.0142204)
Supplement: Supplementary file 6 [file pone.0142204.s001.doc]

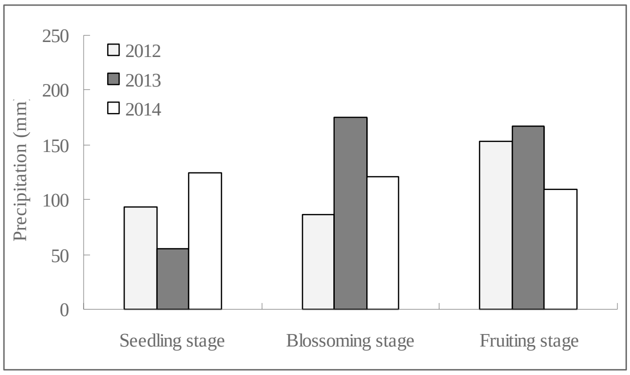

250
200
150
100
50
0
Precipitation (mm
2012
2013
2014
Seedling stage
Blossoming stage
Fruiting stage
