# Supplementary material for: The Effects of Saline Water Drip Irrigation on Tomato Yield, Quality, and Blossom-End Rot Incidence --- A 3a Case Study in the South of China
Source: PLoS One. 2015 Nov 5;10(11):e0142204. doi: 10.1371/journal.pone.0142204 (PMC4634986; doi:10.1371/journal.pone.0142204)
Supplement: Supplementary file 7 [file pone.0142204.s002.doc]

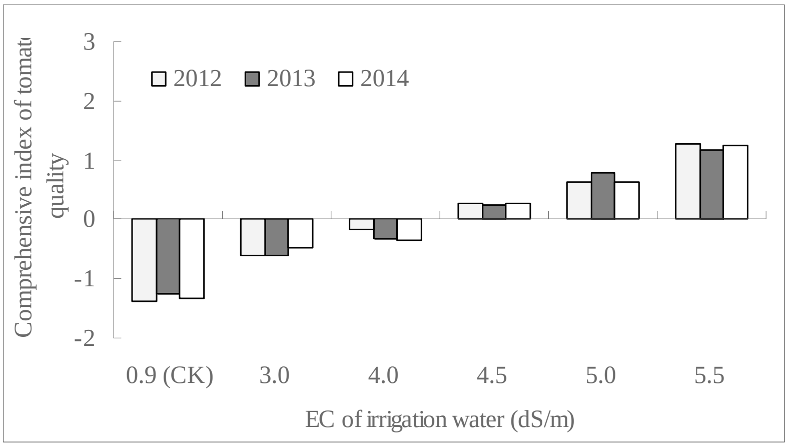

Comprehensive index of tomato quality
3
2
1
0
-1
-2
2012
2013
2014
0.9 (CK)
3.0
4.0
4.5
5.0
5.5
EC of irrigation water (dS/m)
