# Supplementary material for: The Effects of Saline Water Drip Irrigation on Tomato Yield, Quality, and Blossom-End Rot Incidence --- A 3a Case Study in the South of China
Source: PLoS One. 2015 Nov 5;10(11):e0142204. doi: 10.1371/journal.pone.0142204 (PMC4634986; doi:10.1371/journal.pone.0142204)
Supplement: Supplementary file 8 [file pone.0142204.s003.doc]

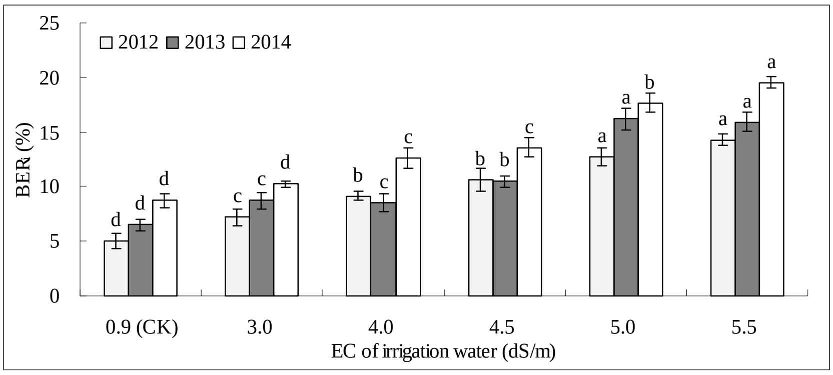

BER (%)
2012 2013 2014
EC of irrigation water (dS/m)
0.9 (CK)
3.0
4.0
4.5
5.0
5.5
0
5
10
15
20
25
d d d
c c d
b c c
b b c
a a b
a a a
